# A cell size- and cell cycle-aware stochastic model for predicting time-dynamic gene network activity in individual cells

**DOI:** 10.1186/s12918-015-0240-5

**Published:** 2015-12-09

**Authors:** Ruijie Song, Weilin Peng, Ping Liu, Murat Acar

**Affiliations:** Interdepartmental Program in Computational Biology and Bioinformatics, Yale University, 300 George Street, Suite 501, New Haven, CT 06511 USA; Systems Biology Institute, Yale University, 840 West Campus Drive, West Haven, CT 06516 USA; Department of Molecular Cellular and Developmental Biology, Yale University, 219 Prospect Street, New Haven, CT 06511 USA; Department of Physics, Yale University, 217 Prospect Street, New Haven, CT 06511 USA

**Keywords:** Stochastic modeling, Galactose network, Noise in gene expression, Single cell data, Yeast

## Abstract

**Background:**

Despite the development of various modeling approaches to predict gene network activity, a time dynamic stochastic model taking into account real-time changes in cell volume and cell cycle stages is still missing.

**Results:**

Here we present a stochastic single-cell model that can be applied to any eukaryotic gene network with any number of components. The model tracks changes in cell volume, DNA replication, and cell division, and dynamically adjusts rates of stochastic reactions based on this information. By tracking cell division, the model can maintain cell lineage information, allowing the researcher to trace the descendants of any single cell and therefore study cell lineage effects. To test the predictive power of our model, we applied it to the canonical galactose network of the yeast *Saccharomyces cerevisiae*. Using a minimal set of free parameters and across several galactose induction conditions, the model effectively captured several details of the experimentally-obtained single-cell network activity levels as well as phenotypic switching rates.

**Conclusion:**

Our model can readily be customized to model any gene network in any of the commonly used cells types, offering a novel and user-friendly stochastic modeling capability to the systems biology field.

**Electronic supplementary material:**

The online version of this article (doi:10.1186/s12918-015-0240-5) contains supplementary material, which is available to authorized users.

## Background

It is well established that gene expression can vary significantly from cell to cell, even in the same clonal population [1–4], in no small part due to the stochastic nature of transcription events in any single cell [5]. Much work has been done to computationally model gene expression networks, including the well-characterized galactose utilization network (GAL network) in yeast. Many of those models [6–9], however, are deterministic models and therefore could provide only limited insights on what happens at the single-cell level. The shortcomings of this approach is demonstrated by previous work [10] that showed that stochastic noise could generate bimodality in a system whose deterministic models predict no bistability.

Therefore, to understand these inherently stochastic processes at the single-cell level, stochastic models are all but required. Many such models in recently published works [10, 11], however, suffer from several deficiencies. These models usually do not take into account variations in rates of transcription, translation, or cell growth among isogenic cells. Nor do they take into account the cell cycle, whose impact on transcription has recently been suggested to be capable of accounting for most of the noise in gene expression [12]. In essence, they model a cell that is stuck indefinitely in the G1 phase of the cell cycle. Such approximations could be acceptable if the simulations lasted for a time period much shorter than the duration of the cell cycle, but they would be questionable for longer simulation durations.

Here we introduce a detailed stochastic model of gene network activity that can be applied to any eukaryotic gene network. The model takes into account real-time changes in cell volume and cell cycle, and it can time-dynamically track the lineage of individual cells while each cell changes its size and gene expression content. To show the efficiency and predictive power of our model, we applied it to the well-characterized GAL network. Using the yellow fluorescent protein (YFP) driven by the GAL1 promoter (P_GAL1_-YFP) as a reporter, we experimentally quantified its expression levels from single cells at two different time points and used these data for fitting, followed by model predictions without any fit parameters. Our model could effectively capture several details of these single cell expression distributions as well as phenotypic switching rates by using a minimal set of free parameters.

## Results

### Modeling cell volume growth and division

**Fig. 1:**
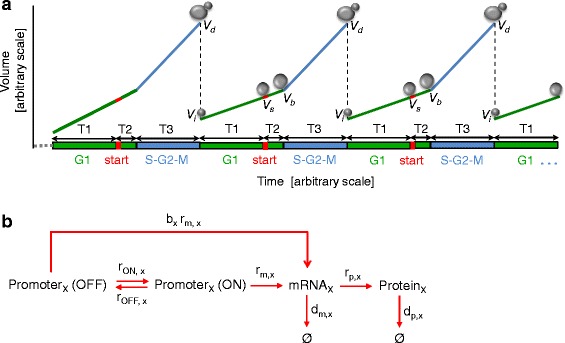
The two modules of the stochastic single-cell model. **a**. Illustration of a model of cell growth with asymmetric division [13]. The cell grows at different rates in the cell cycle stages of G1 and S-G2-M, with the volume increase in S-G2-M being primarily attributable to the daughter compartment. **b**. The seven stochastic reactions associated with each gene in the network and the rate constants. The promoter switches between ON and OFF states. The ON state promoter is transcribed to produce mRNA, while the OFF state promoter also has some basal, leaky transcription. mRNA is translated to produce protein; and both mRNA and protein can be degraded

Our stochastic single-cell model consists of two interrelated modules. The first module models the dynamics of cell volume growth and division. For this, we modeled cell growth and division in the asymmetrically growing budding yeast *S. cerevisiae*. Based on a previous experimental characterization [13], we divided the cell cycle into two stages, one consisting of G1 and the other consisting of S/G2/M. As illustrated in Fig. 1a, the cell volume grows linearly in both stages but at different rates. It was previously shown [13] that the volume at which *start* is reached (leading to the ending of G1 and entry into S after a brief time period) is linearly related to the growth rate in G1, while the volume growth in S/G2/M is mostly attributable to the bud, and determines the size of the daughter cell.

The cell cycle is divided into two stages (G1 and S/G2/M) and three time blocks (T1, T2, T3) (Fig. 1a). T1 consists of the beginning of G1 until *start*; T2 from *start* until the end of G1; and T3 consists of the entire second stage of the cell cycle (S/G2/M). The value of T1 for each cell follows the following equation:

$$ T1= \min \kern0.28em \left(T{1}^{\prime },\frac{V_s-{V}_0}{r_1}\right),\kern1.74em {V}_s=k{r}_1+b $$

where *T1'* provides a lower bound to the length of *T1*, *V*_*0*_ is the volume of the cell at the beginning of the cell cycle, *V*_*s*_ is the volume of the cell at *start*, *r*_*1*_ is the rate of volume growth in G1, and *k* and *b* are model parameters relating *r*_*1*_ to *V*_*s*_. The model parameters consist of the mean and standard deviations of the initial volume of the starting cells (*V*_*i*_), the growth rate in G1 (*r*_*1*_), the overall growth rate in S/G2/M (*r*_*2*_), the mother compartment’s growth rate in S/G2/M (*r*_*2m*_), the minimum length of T1 (*T1'*), the duration from *start* to S phase entry (T2), and the duration of S/G2/M (*T3*), each of which is assumed to follow a normal distribution, along with *k* and *b.*

At each cell division, daughter cells inherit a certain degree of the parameters of their parent. The exact level of inheritance is described by an additional model parameter, *c*, such that for a given parameter *p,*

$$ {p}_{daughter}=c\;{p}_{parent}+\left(1-c\right){p}_{fresh} $$

where *p*_*fresh*_ is a value sampled from the distribution of *p*.

### Modeling the activity of an N-component gene network

The second module of our model is a stochastic model of an *N*-component gene network. The genes composing the network are under the control of a master transcription factor that controls the activation rate of the network promoters. We denote the genes inside the network by *G*_*1*_*, G*_*2*_*, …, G*_*N*_, and have the activity of the network be reported by a fluorescent reporter gene denoted by G_0_. The reporter gene’s activity is determined by the network’s activity. We model the promoter of each gene to switch between two states, OFF and ON, with full-strength transcription only occurring in the ON state. We denote the mRNA corresponding to *G*_*x*_ (where *x = 0, 1, …, N)* as *R*_*x*_, and the protein as *P*_*x*_. The number of copies of promoters of gene *G*_*x*_ in OFF (ON) state is denoted as *PR*_*OFF,x*_ (*PR*_*ON,x*_). Then, for each gene *G*_*x*_ including the reporter, we construct a set of seven stochastic reactions, as illustrated in Fig. 1b. For a cell with volume *V*, the reaction rates for each reaction are: 

Promoter activation: *r*_*ON*, *x*_ *PR*_*OFF*,*x*_
Promoter inactivation: *r*_*OFF*, *x*_ *PR*_*ON*, *x*_
mRNA synthesis from inactive promoter: ${r}_{m,x}\ {b}_x\ P{R}_{OFF,x}\ \frac{V_{ref}}{V}$
mRNA synthesis from active promoter: ${r}_{m,x}\ P{R}_{ON,x}\ \frac{V_{ref}}{V}$
mRNA degradation: $$ {d}_{m,x}\ {R}_x\ \frac{V_{ref}}{V} $$
Protein synthesis: $$ {r}_{p,x}\ {R}_x\ \frac{V_{ref}}{V} $$
Protein degradation: $$ {d}_{p,x}\ {P}_x\ \frac{V_{ref}}{V} $$

* V*_*ref*_ is a constant scaling factor equal to the average volume of the entire population of cells. We introduced it to make the value of reaction parameters more comparable to experimental measurements. The stochastic reactions are governed by the parameters *r*_*OFF,x*_, *r*_*ON,x*_, *r*_*m,x*_, *b*_*x,*_*r*_*p,x*_, *d*_*m,x*_, and *d*_*p,x*_. The parameter *r*_*ON,x*_ is determined by the following equation:

$$ {r}_{ON,x}={r}_x\ F\left(\left[ inducer\right],\ \left[{P}_1\right],\ \left[{P}_2\right],\dots, \left[{P}_N\right]\right) $$

where *r*_*x*_ is the maximum activation rate of the promoter of *G*_*x*_. *F* is a function relating the concentrations of the inducer and *P*_*1*_*, P*_*2*_*, …, P*_*N*_ to the overall activity of the network, and is called the *functional form* of the network.

We further opted to use as a model parameter not the promoter inactivation rate *r*_*OFF,x*_*,* but instead the fraction of time a promoter spends active when fully induced. We define this fraction, *f*_*x*_, as

$$ {f}_x=\frac{r_x}{r_x+{r}_{OFF,x}} $$

Both *f*_*x*_ and *r*_*x*_ are model parameters for all *x = 0, 1, …, N*.

Further, in our actual model, we use not the actual mRNA synthesis rate per promoter *r*_*m,x*_, but the observed mRNA synthesis rate *r'*_*m,x*_. The two are related by the equation

$$ {r}_{m,x}^{\prime }={r}_{m,x}\ {f}_x+{b}_x{r}_{m,x}\left(1-{f}_x\right) $$

This implementation reflects that, even in fully induced cells, the promoters are only being transcribed at maximum rate a portion (*f*_*x*_) of the time. Similarly, we use the observed basal expression level *b'*_*x*_, instead of the actual ratio between OFF-state and ON-state transcription rates *b*_*x*_. The two are related by the equation

$$ {b}_x^{\prime }\ {r}_{m,x}^{\prime }={b}_x\ {r}_{m,x} $$

To summarize, for each network component (and the reporter), there are seven stochastic reactions described by seven parameters: *r*_*x*_, *f*_*x*_, *r'*_*m,x*_, *b'*_*x*_, *r*_*p,x*_, *d*_*m,x*_, and *d*_*p,x*_.

### Combining the gene network activity model with the cell growth and division model

Next, we incorporated cell volume growth and division into our stochastic gene network model by including cell volume as an additional stochastic reaction. The rate at which the reaction fires is determined by the current cell volume growth rate as calculated by the volume model, and each time the stochastic reaction fires, the cell volume is increased by a small, fixed amount determined by the volume model. This change in cell volume in turn changes the rates of the stochastic reactions.

Our model also takes into account the changes in the number of copies of the genetic material during cell cycle. During DNA replication, when an active promoter replicates into two promoters, we assume that both promoters remain active. Similarly, an inactive promoter is assumed to replicate into two inactive promoters during DNA replication. Further, during cell division, we distribute the mRNA and protein contents between mother and daughter cells in accordance with a binomial distribution whose probability *p* is equal to the ratio of the volume of the daughter cell to the total volume.

### Simulations using the combined stochastic model

Using this single-cell level model, we simulated populations of cells for 22 h and 5 h, which were also the time periods we used for growing the cells in our experiments.

**Fig. 2:**
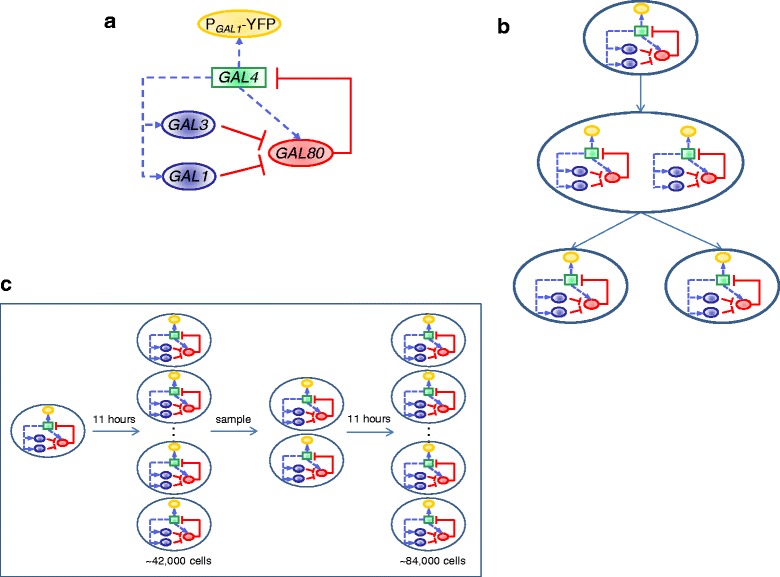
Overview of the GAL network and the simulation process. **a**. The GAL network in *S. cerevisiae* with its activity reported by a P_*GAL1*_-YFP reporter. Transcription of *GAL1*, *GAL3*, *GAL80*, and the reporter are controlled by the transcription factor Gal4p. Gal80p represses Gal4p and is in turn repressed by the inducer Gal3p and Gal1p in the presence of galactose. **b**. As a cell grows, both the volume and the number of gene and network copies will change. **c**. The simulation and sampling process for the 22-h simulation. Each cell in the figure represents 1000 cells; the initial population is simulated for 11 h, resulting in a population of ~42,000 cells; from this population 2000 cells are sampled and simulated for an additional 11 h to produce the final population of cells

However, due to exponential growth of the population size, with any reasonable size for the initial population of cells, it is impractical to simulate all of its descendants for 22 h. Hence, we added a sampling step (Fig. 2c). For the 22 h simulation, we started from an initial population of 1000 cells, ran the simulation for 11 h and randomly sampled 2000 cells from the resulting population of ~42,000 cells, and then simulated those cells for another 11 h for a final population of ~84,000 cells.

For the 5 h simulation, to minimize the effect of the initial state on the result of the simulation (due to the relatively shorter time period compared to 22 h), an initial population of 20,000 cells were simulated for 5 h in basal conditions, a sample of 20,000 cells were taken, the inducer was introduced to the system, and the sampled cells were simulated for an additional 5 h.

**Fig. 3:**
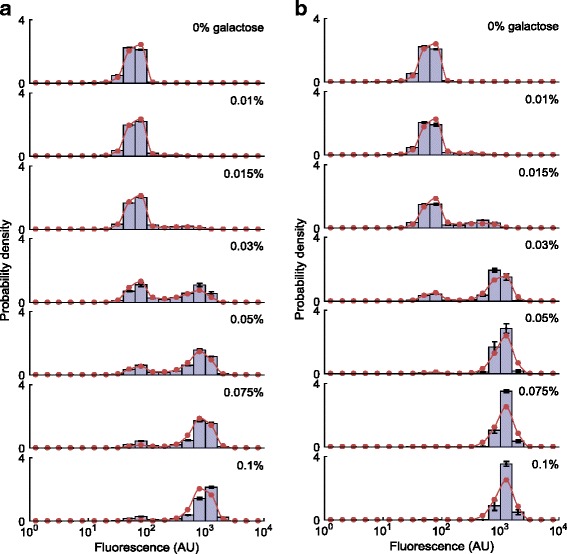
Comparison between fitting results and experimental measurements. Comparison of the fitting results and experimentally-measured single-cell fluorescence distributions at the specified galactose concentrations, for the 5-h (**a**) and 22-h (**b**) experiments. Bars indicate experimentally measured distributions. Error bars represent SEM. Red dots indicate results produced by the simulation using parameters obtained from fitting

We repeated this simulation and sampling process for several different inducer concentrations (Fig. 3). The output of the simulation with a given set of parameters and inducer concentration was a set of reporter protein counts in the final population of cells, which we converted to simulated fluorescence measurements by using a fitting procedure (Methods)*.*

### Application of the model to the canonical GAL network in yeast

We tested the efficiency and predictive power of our model by applying it to the GAL network of the yeast *S. cerevisiae.* The GAL network is arguably the most suitable gene network to test our model due to the network’s well-characterized [14–18] nature in terms of its components and their interaction topology. Choosing a canonical gene network allows one to study principles affecting gene network activity that are broadly applicable to eukaryotic cells.

The activity of the GAL network is governed by a master transcription factor Gal4p that binds to the network promoters to activate their transcription (Fig. 2a). Previous work has shown that two additional regulatory proteins (Gal80p and Gal3p), as well as the galactokinase Gal1p, play key roles in setting the activity of the GAL network [19–22]. Gal80p is a repressor that binds to Gal4p to repress it, Gal3p is an inducer that binds to Gal80p in the presence of galactose and relieves the repression, and Gal1p (which is highly homologous to Gal3p) is also an inducer, although significantly weaker than Gal3p.

The dual positive feedback loops formed by GAL1 and GAL3, together with the inherent nonlinearities of the GAL network, result in a bimodal expression profile [15, 19, 23]. The functional form that we used for the GAL network (see Methods) captures all of these interactions.

For the parameters governing the dynamics of the GAL gene network activity, we fixed several of them in ranges described in published literature. These included the rates of RNA synthesis and decay [24], translation [10, 25, 26], and protein degradation [26], as well as basal transcription levels for the GAL network promoters [27]. Since inside our model a scaling parameter, subject to fitting, is applied to each protein, we do not expect inaccuracies in these values to have substantial effects on the predictive power of our model.

To simulate global transcriptional noise in cells, we applied a random perturbation to each rate parameter for each individual cell. Inside each cell, the rate parameters for each process (e.g., transcription) are perturbed by the same fraction for all network components, to reflect that these perturbations are caused by global noise extrinsic to the particular gene.

The only free parameters that we used in our model were the ones governing the transitions of the network promoters between the OFF and ON states, and the parameters setting the scale of action for network proteins, which were determined by sweeping over discrete values followed by fitting to experimental data. For this, the Monte Carlo simulation generated a distribution of reporter protein levels in the final population of cells and this was then fitted to experimental measurements obtained from a yeast strain carrying the P_GAL1_-YFP reporter construct. The GAL1 promoter is a faithful reporter of the network activity, as it is bound and activated by the Gal4 proteins.

To experimentally obtain the P_GAL1_-YFP expression distributions at the single-cell level, we induced the yeast cells for 5 and 22 h in media containing seven different galactose concentrations. Using a flow cytometer, we then measured the YFP expression levels from ~10,000 cells and obtained the expression histograms depicted in Fig. 3. We then binned the expression levels to obtain 20 regions on each histogram, and fitted our model to these experimental results obtained at 5 and 22 h, producing the fit results depicted in Fig. 3. The parameter values obtained from the fitting procedure are shown in Additional file 1: Table S2. As can be seen, the fit results are in good agreement with the experimentally obtained values.

**Fig. 4:**
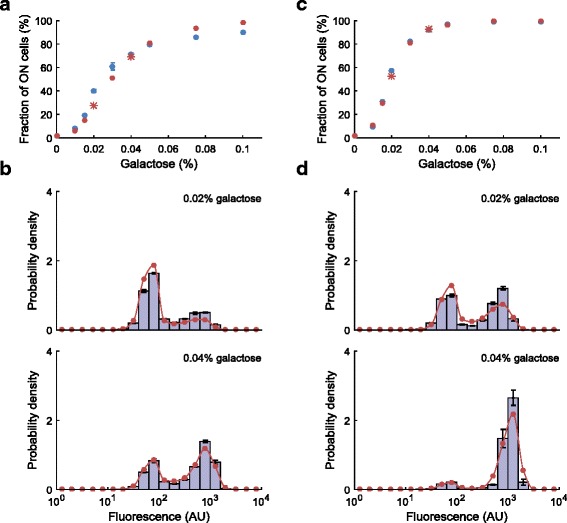
The predictive power of the model. **a**, **c**, Fraction of ON cells as a function of galactose concentration for 5-h (**a**) and 22-h (**c**) experiments: experimental (blue dots) vs. fitted (red dots) and predicted (red stars). Error bars represent SEM. **b**, **d**, Single-cell fluorescence distributions of *S. cerevisiae* with a P_GAL1_-YFP reporter are measured at two additional galactose concentrations (0.02 % and 0.04 %) after 5 h (**b**) and 22 h (**d**) of induction and compared against predictions from the model fitted without these data. Bars represent experimental measurements; error bars represent SEM. The model predictions without any fitting are in red

To test the predictive power of our model, we performed additional experimental measurements in two more media conditions containing 0.02 % and 0.04 % galactose, for both experimental durations (5 and 22 h), and compared the experimental measurements with the predictions of the model without using any free parameters. We selected these concentrations because, as shown in Fig. 4a and c, they are in the ‘linear’ range in which changes in inducer concentration causes significant changes in the fraction of ON cells. As shown in Fig. 4, the predictions of our model for those concentrations are in good agreement with the experimental observations, both in terms of the fraction of ON cells and in terms of the single-cell fluorescence distributions.

### Tracking the lineage-specific changes in cell size and protein content in real time

**Fig. 5:**
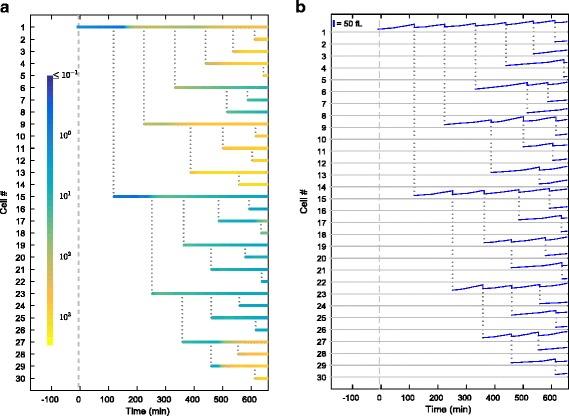
Demonstration of the model’s capability to track individual cells and lineages. Traces of reporter protein concentration (**a**) and cell volume (**b**) in descendants of an individual cell (labeled cell #1) for the first 660 min of the simulation. Protein concentrations are in arbitrary units. Each horizontal line represents a cell. Dotted gray lines indicate cell division. The scale bar in (**b**) is 50 fL. Galactose (0.03 %) is introduced into the system at t = 0

Our model also allows users to track lineages of individual cells and how their size and gene expression contents change as a function of time. In Fig. 5, we selected a representative cell from a simulation run at 0.03 % galactose using the parameters obtained from the fitting procedure described above, and tracked the single-cell reporter content (Fig. 5a) and cell size (Fig. 5b) of all of its descendants born during the first 660 min of the simulation. Our model also allows users to perform more detailed analyses such as comparing the gene expression and cell size characteristics of the descendants of different cells.

**Fig. 6:**
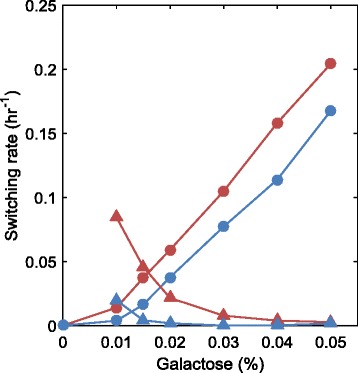
Comparison between experimentally measured and model predicted phenotypic switching rates. Experimentally measured (blue) and model predicted (red) phenotypic switching rates, as a function of galactose concentration. Circles indicate OFF-to-ON switching rates; triangles indicate ON-to-OFF switching rates

A final demonstration for the predictive power of our model was made by experimentally measuring the phenotypic switching rates between the OFF and ON states of the GAL network, followed by predicting the rates by our model without using any additional fit parameters. Using a detailed log produced by the simulation, each cell was classified as either ON or OFF at each time point recorded based on the number of reporter proteins in the cell. Then, the rates at which OFF cells switch to the ON state and ON cells switch to OFF state were calculated. As shown in Fig. 6, the results of our model are generally consistent with the estimated phenotypic switching rates extracted by applying a simple two-state model (without any free parameters) to experimental data obtained from two different initial conditions [28] (see Additional file 1: Note S2). The experimental method we used to extract the switching rates is an alternative to the more direct but prohibitively time-taking method of microscopically tracking thousands of individual cells for multiple hours in the presence of cell-crowding and focusing issues. We expected that our experimental method would underestimate the switching rates and indeed the values we extracted from data were lower than the ones predicted by the simulation. This expectation was due to the fact that our method would not count the phenotypic switching events if, for example, a cell switched between the two phenotypic states for an even number of times.

## Discussion

Using computational analysis to guide experimental testing has significant advantages over the traditional purely experimental approach. By using the computational predictions as a guide, the researcher can avoid the inefficiencies associated with a purely experimental approach, while still producing results that can be actually tested and confirmed in a biological system.

The volume of a cell and the cell cycle stage it goes through can have significant impact on the activity of a gene network in that cell. Doubling the volume effectively halves the gene network dosage, which can significantly change the network activity level for non-dosage-compensated networks [14, 29]. Similarly, it has been suggested that the cell cycle, and the transcription changes it causes, is a major contributor to gene expression noise observed in a population of cells [12]. To fully understand the complex interactions at play, one needs a model that accounts for both the cell volume and cell cycle, and the network itself.

Here we present such a single-cell level stochastic model and demonstrate its predictive power by using the GAL network in *S. cerevisiae.* We validated our model by comparing its predictions for single-cell gene expression distributions with experimental results obtained at different galactose induction levels that were not used to select model parameter values. Our model is also able to generate detailed single-cell level lineage-specific time-course data for gene expression, cell volume, and cell division. Using this data, we calculated the phenotypic switching rates for the cells in the simulation and saw that the results were in reasonably good agreement with the switching rate estimates obtained from additional experiments.

We note that our volume model results in a steadily increasing cell volume as the cells age. This is consistent with experimental observations [30, 31] on *S. cerevisiae* cells, whose volume indeed increase steadily until they reach a relatively constant maximal volume and enter senescence. Our volume model does not attempt to capture senescence, for two reasons. First, the time scale of our simulations (22 h, or ~11 generations) is well below the average replicative lifespan for yeast cells (24–29 generations [31, 32]), so that few cells would be expected to reach such a state. Second, in an exponentially growing population of cells, the fraction of old cells is extremely small due to geometric distribution (leading to the population composition of 50 % newborn cells, 25 % one-generation old cells, and so on), and would not appreciably affect our results.

## Conclusion

In this paper, we present a single-cell level stochastic model that accounts for the cell volume and the cell cycle in addition to the gene network it models, and demonstrate its predictive power by using the GAL network in *S. cerevisiae.* Our model can easily be adapted for other gene networks and other cell types, and can also be easily extended in other ways. For instance, researchers working with different cell types (e.g., mammalian cells, or fission yeast) need only create a volume model reflecting the size-control mechanism in those cells, without having to reinvent the gene network part of the model. As another example, by having the gene network part of the model affect the volume growth and cell division rates via a fitness function, one can easily modify the model to perform *in silico* evolution experiments to track and understand how gene networks evolve time dynamically.

## Methods

### Strain construction

We used a BY-background haploid wild-type *S. cerevisiae* strain carrying the P_*GAL1*_-YFP reporter integrated in its *ho* locus. For this, *KpnI − P*_*GAL1*_ *− BamHI* and *BamHI − YFP − EcoRI* fragments were cloned into a plasmid upstream of the *CYC1* transcriptional terminator. The plasmid also carried the *P*_*TEF1*_-*HIS5* marker positioned to the left of the P_*GAL1*_-YFP reporter. Using this plasmid as a template together with 5’-3’ primers having 60 bp-long homology to the *ho* locus, the [*P*_*TEF1*_-*HIS5* + P_*GAL1*_-YFP] region of the plasmid was PCR amplified and then transformed into yeast. The P_*GAL1*_ promoter sequence corresponds to the 668 base-pair region directly upstream of the start codon of the *S. cerevisiae GAL1* gene. The genetic composition of the strain we used is: *MATα, his3Δ, leu2Δ, LYS2, met15Δ, ura3Δ, ho::HIS5-P*_*GAL1*_*-YFP.*

### Growth conditions and media

Cultures were grown in synthetic dropout media with the appropriate amino-acid supplements. During the overnight growth period (22 h in 30 °C shaker), 0.1 % mannose was used as a non-inducing carbon source. The overnight growth period was followed by the induction period (5 or 22 h in 30 °C shaker), with cultures containing 0.1 % mannose and 0–0.1 % galactose as carbon sources. 0.1 % mannose was used as a background carbon source ensuring similar growth rates across different galactose concentrations. After the induction period, the expression distributions of approximately 10,000 cells were measured by a flow cytometer (FACS-Verse; Becton Dickinson). The OD_600_ values at the end of the overnight and induction periods were kept low (OD_600_ ~ 0.1) to prevent nutrient depletion. The culture volumes were 5 ml during both the overnight growth and induction periods.

For switching-rate measurement experiments, the overnight growth media contained either [0.1 % mannose, for OFF history] or [0.1 % mannose and 0.1 % galactose, for ON history] as carbon sources, so that we would obtain different initial conditions at the end of the overnight period. Following the overnight growth, OFF and ON history cultures were separately induced for 22 h in the same media containing 0.1 % mannose and 0–0.1 % galactose. After the overnight and induction periods, the expression distributions of approximately 10,000 cells were measured by a flow cytometer. The fractions of ON cells at the beginning and end of the induction period were quantified and used in extracting the phenotypic switching rates as described in Additional file 1: Note S2.

### Setting parameter values for the cell-growth and division module

The asymmetric volume model described has the following parameters: means and standard deviations of *V*_*i*_, *r*_*1*_, *r*_*2*_, *r*_*2m*_, *T2, T1’*, and *T3*, along with the parameters *k*, *b,* and *c.* The values of all parameters except *c* were taken from [13], which performed time-course microscopic volume measurement experiments on the same strain background as the one we used; the value of *c* was fixed at 0.25. Additional file 1: Table S1 shows the values of the parameters we used for the cell growth and division module.

### Software for simulations and fitting

All code used for simulation and fitting is custom-written in C++. Random numbers for the simulation are generated using the TRNG library [33]. Fitting is performed using the NLopt library [34].

### Simulations of the combined stochastic model

For a given set of model parameters, the simulation was performed using a modified version [35, 36] of the well-established Gillespie algorithm [37]. We simulate a population of cells, with the model parameters for each cell sampled from a normal distribution with mean equal to the parameter provided and standard deviation equal to 10 % of the mean. The age of each cell at t = 0 is sampled from an exponential distribution with mean equal to the average doubling time of the strain (120 min). The initial state of each cell was set according to the steady state basal levels calculated from its parameter values.

For the 22 h simulations, we started from an initial population of 1000 cells. Inducer is introduced at t = 0. The simulation is run for 11 h, and a sample of 2000 cells is taken from the resulting population of ~42,000 cells, to be simulated for another 11 h, for a final population of ~84,000 cells.

For the 5 h simulations, we started from an initial population of 20,000 cells, which is simulated for 5 h at basal conditions (no inducer). A sample of 20,000 cells is taken from the resulting population of cells, the inducer is introduced, and the sample is simulated for another 5 h.

The output of the simulation is a set of reporter protein counts in *n* cells *R = {R*_*1*_*, R*_*2*_*, …, R*_*n*_*}.* To match this output to the experimentally observed fluorescence data, we performed the fitting procedure as described below in the section titled “Fitting procedure for fluorescence”.

### Fitting the combined stochastic model to single-cell gene expression distributions

We use the following functional form to represent the activity of the GAL network:

$$ F=\frac{1}{1+{\left(\frac{S_{80}\left[ Gal80p\right]}{1+{\left({S}_3g\left[ Gal3p\right]+{S}_1g\left[ Gal1p\right]\right)}^{\alpha }}\right)}^{\beta }} $$

where *S*_*3*_, *S*_*1*_, *S*_*80*_, *α* and *β* are model parameters. We note that this functional form is not generic, but it can be derived from the molecular interactions of the network components, as shown in Additional file 1: Note S1.

We set α = 1 based on previous work [14], in which case the functional form becomes

$$ F=\frac{1}{1+{\left(\frac{S_{80}\left[ Gal80p\right]}{1+{S}_3g\left[ Gal3p\right]+{S}_1g\left[ Gal1p\right]}\right)}^{\beta }} $$

$$ \approx \frac{1}{1+{\left(\frac{S_{80}\left[ Gal80p\right]}{S_3g\left[ Gal3p\right]+{S}_1g\left[ Gal1p\right]}\right)}^{\beta }}\kern1.25em \mathrm{when}\ {S}_3g\left[ Gal3p\right]+{S}_1g\left[ Gal1p\right]\gg 1 $$

When *S*_3_*g*[*Gal*3*p*] + *S*_1_*g*[*Gal*1*p*] ≫ 1, proportionally changing the values of *S*_*3*_, *S*_*1,*_ and *S*_*80*_ does not affect the value of *F*. Accordingly, we fixed *S*_*80*_ at the arbitrary number 4500 and fitted *S*_*3*_ and *S*_*1*_ only.

We fixed *r'*_*m,x*_, *r*_*p,x*_, *b*_*x*_, *d*_*m,x*_, and *d*_*p,x*_ for the reporter (P_*GAL1*_-YFP) and all network components (GAL1, GAL3, and GAL80) based on values reported in literature (see Additional file 1: Table S3). The parameters to be fitted consist of *r*_*x*_ and *f*_*x*_ for the reporter and network components, and *S*_*3*_, *S*_*1*_ and *β* in the functional form of the network, for a total of nine parameters (as the reporter and the GAL1 gene share the same promoter, they are assumed to have the same *r*_*x*_ and *f*_*x*_ values).

We performed sweeps over a wide range of possible parameter values and selected initial values of the parameters for fitting so that they yielded bimodal fluorescence distributions similar to the behavior of the GAL network. The fitting was performed using the well-known Nelder-Mead algorithm [38–40]. For each set of parameters, the simulation as described above is repeated a number of times (denoted N_R_). Each repeat consists of a 5-h simulation and a 22-h simulation. The score of the repeat is obtained as described below; the mean of the scores of each repeat is taken as the score for the set of parameters. The fitting algorithm was first run for 24 h (wall-clock time) with N_R_ = 32, and then for an additional 48 h (wall-clock time) with N_R_ = 128.

### Fitting procedure for fluorescence

Given a set of reporter protein counts in *n* cells *R = {R*_*1*_*, R*_*2*_*, …, R*_*n*_*},* we generate a set of background fluorescence values *B = {B*_*1*_*, B*_*2*_*, …, B*_*n*_*}*, where each *B*_*i*_ is sampled from a normal distribution whose parameters are determined using a population of uninduced cells (μ = 61, σ = 17).

Given *R, B,* and a particular reporter-to-fluorescence conversion factor *c*, we define the likelihood function as follows. For each cell *i* = *{1*, …, *n}*, we let the cell’s total fluorescence be *F*_*i*_ *= cR*_*i*_ *+ B*_*i*_*.* Then, we generate a histogram of *log*_*10*_*(F*_*i*_*),* with bins [0, 0.2), [0.2, 0.4), …, [3.8, 4), normalized to total area of 1. Having *H*_*a, b*_ denote the height of the bin [*a, b*), we define *pdf(g) = max(0.0001, H*_*a,b*_*)*, where [*a,b*) is the bin containing *log*_*10*_*(g).* Then, given the known experimental observations of *n* cells with fluorescence *E*_*1*_*, E*_*2*_*, …, E*_*n*_, the likelihood function is given by $L\left(R,B,c\right) = {\displaystyle \prod_{i=1}^n}pdf\left({E}_i\right)$.

As the simulation as described above generates a set of *R*’s (one for each inducer concentration)*,* we generate a set of *B*’s, one for each *R*. The actual likelihood function *L(c)* is the product of the values of *L(R, B, c)* described above for each pair of R and B. We use the Nelder-Mead algorithm to find the value *c* that maximizes the value of *L(c)* (or minimizes the value of *-log(L)*). The resulting maximized value of *L* is the likelihood, and the corresponding value *c* is the optimal reporter-to-fluorescence conversion factor.

During fitting for the network model, the likelihood function is computed by multiplying the likelihood functions for the 5-h and the 22-h simulations computed as described above, and the fitting procedure seeks the value *c* that maximized the value of the combined likelihood function. The fluorescence fitting procedure is repeated a number of times for each repeat of the simulation (32 times for the first stage of the fitting, and 120 times for the second stage), and the mean of the obtained values of *L* is used as the score of the run.

### Availability of supporting data

The data sets supporting the results of this article are included within the article and its additional file.

## Additional file

Additional file 1:
**Contains Notes S1 (derivation of the functional form of the GAL network) and S2 (description of phenotypic switching rate characterization), and Tables S1, S2 and S3 (lists of model parameters and their values).** (PDF 511 kb)

## Abbreviations

GAL network: galactose utilization network
YFP: yellow fluorescent protein
