# Supplementary material for: A cell size- and cell cycle-aware stochastic model for predicting time-dynamic gene network activity in individual cells
Source: BMC Syst Biol. 2015 Dec 9;9:91. doi: 10.1186/s12918-015-0240-5 (PMC4673848; doi:10.1186/s12918-015-0240-5)
Supplement: Additional file 1: — Contains Notes S1 (derivation of the functional form of the GAL network) and S2 (description of phenotypic switching rate characterization), and Tables S1, S2 and S3 (lists of model parameters and their values). (PDF 511 kb) [file 12918_2015_240_MOESM1_ESM.pdf]

A cell size- and cell cycle-aware stochastic model for predicting time-dynamic gene network activity in individual cells

SUPPLEMENTARY INFORMATION

Ruijie Song[1, 2], Weilin Peng[2, 3], Ping Liu[2, 3], and Murat Acar[1, 2, 3, 4, †]

[1] Interdepartmental Program in Computational Biology and Bioinformatics, Yale University, 300 George Street, Suite 501, New Haven, CT 06511

[2] Systems Biology Institute, Yale University 840 West Campus Drive, West Haven, CT 06516

[3] Department of Molecular Cellular and Developmental Biology, Yale University 219 Prospect Street, New Haven, CT 06511

[4] Department of Physics, Yale University 217 Prospect Street, New Haven, CT 06511

[†] To whom correspondence should be addressed: E-mail: murat.acar@yale.edu

SUPPLEMENTARY NOTES

1. Derivation of the functional form for GAL network activity.

We consider a bimodal network that transitions between the OFF and ON states, in which the total concentrations of the relevant regulatory proteins affect the rate at which the OFF→ON transition takes place:

$$\text{OFF} \underset{h}{\overset{h\rho(x_1, x_3, x_4, x_{80})}{\rightleftharpoons}} \text{ON}$$

The parameter h above represents the timescale at which these transitions take place, while $\rho(x_1, x_3, x_4, x_{80})$ is a function that quantifies how the total concentrations of different GAL proteins (x_1, x_3, x_4, x_{80}) affect the OFF→ON transition rate.

We use a functional form to express the activity of the network as a fraction of the maximum activity. This functional form can be derived from the interactions of the network components as follows. Gal4p is the constitutively expressed main transcriptional activator of the network; its interaction with a GAL network promoter can be represented as

$$\rho = \left(\frac{x_4^*}{K_4}\right)^\eta$$

where K_4 represents the typical concentration scale of the interaction, $\eta > 0$ denotes the effective nonlinearity of the Gal4p-promoter interaction, and x_4^* is the active concentration of Gal4p which is not bound by Gal80p and can therefore freely activate transcription.

As Gal4p is constitutively expressed, its total concentration (denoted by x_4) is constant. Therefore, the maximum possible value of ρ is

$$\rho_{\max} = \left(\frac{x_4}{K_4}\right)^\eta$$

Since the amount of free Gal4p (denoted by x_4^*) should be a decreasing function of the concentration of Gal80p and an increasing function of total Gal4p, we used the following equation to model the Gal4p-Gal80p interaction:

$$x_4^* = \frac{x_4}{1 + \left(\frac{x_{80}^*}{K_{80}}\right)^\beta}$$

where x_{80}^* is the concentration of Gal80p proteins that are not bound by active Gal3p or Gal1p, K_{80} is the scaling parameter, and β is the degree of nonlinearity of the Gal4p-Gal80p interaction.

Gal1p and Gal3p are close homologs and inhibit Gal80p via the same mechanism. Since the amount of x_{80}^* should be a decreasing function of active Gal3p proteins (x_3^*) and active Gal1p proteins (x_1^*), we used the following equation to model the Gal80p-Gal3p/Gal1p interaction:

$$x_{80}^* = \frac{x_{80}}{1 + \left(\frac{x_3^*}{K_3} + \frac{x_1^*}{K_1}\right)^{\alpha}}$$

where x_3^* is the concentration of active Gal3p proteins, x_1^* is the concentration of active Gal1p proteins, and the parameter α quantifies the nonlinearity of Gal80p-Gal3p/Gal1p interaction.

Finally, we assume that Gal1p and Gal3p are activated by galactose in a simple linear relation, which is true as long as the amount of galactose is non-saturating:

$$x_1^* = x_1 g$$
$$x_3^* = x_3 g$$

Substitution yields

$$\rho = \left(\frac{\frac{x_4}{K_4}}{1 + \left(\frac{\frac{x_{80}}{K_{80}}}{1 + \left(\frac{x_3 g}{K_3} + \frac{x_1 g}{K_1}\right)^{\alpha}} \right)^{\beta}} \right)^{\eta}$$

Thus, the level of network activity, represented as a fraction of the maximum activity, is

$$F = \frac{\rho}{\rho_{\max}} = \left(\frac{1}{1 + \left(\frac{\frac{x_{80}}{K_{80}}}{1 + \left(\frac{x_3 g}{K_3} + \frac{x_1 g}{K_1}\right)^{\alpha}} \right)^{\beta}} \right)^{\eta}$$

Letting $S_3 = \frac{1}{K_3}$, $S_1 = \frac{1}{K_1}$, $S_{80} = \frac{1}{K_{80}}$, the equation becomes

$$F = \frac{\rho}{\rho_{\max}} = \left(\frac{1}{1 + \left(\frac{S_{80} x_{80}}{1 + (S_3 x_3 g + S_1 x_1 g)^{\alpha}} \right)^{\beta}} \right)^{\eta}$$

Finally, letting $\eta = 1$ produces the functional form we aimed to derive:

$$F = \frac{1}{1 + \left(\frac{S_{80} x_{80}}{1 + (S_3 x_3 g + S_1 x_1 g)^{\alpha}} \right)^{\beta}}$$

2. Experimental and model-based characterization of the phenotypic switching rates.

<u>*Experimental characterization of switching rates*</u>*:* The phenotypic switching rates between the OFF and ON states of the GAL network is extracted from experimental data as previously described[1, 2]. Briefly, the experimentally-obtained number of ON and OFF cells, N_{OFF} and N_{ON}, are entered into the following 2-state model:

$$\begin{cases} \frac{dN_{ON}}{dt} = \gamma N_{ON} - r_{OFF} N_{ON} + r_{ON} N_{OFF} \\ \frac{dN_{OFF}}{dt} = \gamma N_{OFF} + r_{OFF} N_{ON} - r_{ON} N_{OFF} \end{cases}$$

where γ is the growth rate of the cells, and r_{OFF} and r_{ON} are the ON-to-OFF and OFF-to-ON switching rates, respectively. Using these coupled differential equations composed of two equations and two unknowns (r_{OFF} and r_{ON}), it can be shown that the fraction of ON cells, f_{ON}, in such a system, obeys the following equation:

$$f_{ON}(t) = \frac{r_{ON}}{r_{ON} + r_{OFF}} + \left(f_{ON}(t = 0) - \frac{r_{ON}}{r_{ON} + r_{OFF}} \right) e^{-(r_{ON} + r_{OFF})t}$$

Given two different initial states $f_{ON}(t = 0)$ and the corresponding final states after 22 hours $f_{ON}(t = 22h)$, the above equation can be numerically solved for r_{OFF} and r_{ON}. The two different initial states were experimentally obtained by growing yeast cells in [0.1% mannose (OFF history)] and [0.1% mannose + 0.1% galactose (ON history)] respectively for 22 hours, and the fraction of ON cells were measured using FACS. The cells were then grown in induction media, consisting of 0.1% mannose plus nine different concentrations of galactose (from 0% to 0.1%),

for another 22 hours, and the post-induction fraction of ON cells were measured using FACS. A cutoff for ON cells was selected based on FACS measurements performed on uninduced cells (i.e., without galactose). The results obtained from these measurements were used to solve the above equation for r_{OFF} and r_{ON}.

Model-based characterization of switching rates: From the logs of the simulation, the OFF-to-ON and ON-to-OFF switching rates are calculated as follows. At each time point, each cell is classified as being in either ON or OFF state, by comparing its simulated fluorescence level to the OFF cutoff used in the experimental measurements. The number of ON-to-OFF and OFF-to-ON switching events are counted and divided by the total amount of time the simulated cells spent in the ON and OFF states, respectively, to yield the switching rates.

SUPPLEMENTARY TABLES

Supplementary Table 1. Parameters for the cell-growth and division module.

**Parameter**	**Value**	**Unit**
mean and SD of r_1	0.18 ± 0.045	fL min^{-1}
mean and SD of r_2	0.379 ± 0.08103	fL min^{-1}
mean and SD of r_{2m}	0.005 ± 0.018	fL min^{-1}
mean and SD of *T1'*	15.6 ± 8.3616	min
mean and SD of *T2*	19.2 ± 5.9904	min
mean and SD of *T3*	66.9 ± 7.7604	min
mean and SD of V_i	25 ± 5	fL
k	139.2	min
b	11.0	fL
c	0.25	

Supplementary Table 2. Fitted parameters for the gene network module.

**Parameter**	**Fitted value**	**Unit**	**Initial value**	**Lower bound**	**Upper bound**
r_{GAL3}	0.219726	min^{-1}	0.1	0.01	0.5
r_{GAL80}	0.494052	min^{-1}	0.1	0.01	0.5
$r_{GAL1}, r_{PGAL1\text{-}YFP}$	0.213308	min^{-1}	0.1	0.01	0.5
f_{GAL3}	0.99		0.9	0.01	0.99
f_{GAL80}	0.98982		0.9	0.01	0.99
$f_{GAL1}, f_{PGAL1\text{-}YFP}$	0.871232		0.9	0.01	0.99
S_3	31156.9		40000	20000	100000
S_1	1445.82		1000	100	5000
β	3.53303		4	1	10

Supplementary Table 3. Fixed parameters for the gene network module.

Parameter	Value	Unit	References & Notes
$r'_{m, GAL3}$	0.7223	min^{-1}	[Note A]
$r'_{m, GAL80}$	0.1394	min^{-1}	[Note B]
$r'_{m, GAL1}, r'_{m, PGAL1\text{-}YFP}$	1.53828	min^{-1}	[Note C]
$r_{p, GAL3}$	20	min^{-1}	[Note A]
$r_{p, GAL80}$	4	min^{-1}	[Note B]
$r_{p, GAL1}$	20	min^{-1}	[Note D]
$r_{p, PGAL1\text{-}YFP}$	4	min^{-1}	[Note E]
$d_{m, GAL3}$	0.0330	min^{-1}	[3]
$d_{m, GAL80}$	0.02567	min^{-1}	[3]
$d_{m, GAL1}$	0.02666	min^{-1}	[Note F]
$d_{m, PGAL1\text{-}YFP}$	0.02666	min^{-1}	[Note G]
$d_{p, GAL3}$	0.03851	min^{-1}	[4]
$d_{p, GAL80}$	0.00024	min^{-1}	[4]
$d_{p, GAL1}$	0.00048135	min^{-1}	[Note H][5]
$d_{p, PGAL1\text{-}YFP}$	0.00048135	min^{-1}	[Note H]
$b'_{GAL1}, b'_{PGAL1\text{-}YFP}$	0.0018		[6]
b'_{GAL3}	0.07294		[6]
b'_{GAL80}	0.21739		[6]
S_{80}	4500		[Note I]
α	1		[7]
V_{ref}	50	fL	[Note J]

[Note A] Calculated based on a basal level of 721 Gal3p/cell [8], the basal transcription level calculated from [6], the protein half-life from [4], and a translation rate of 20/mRNA/min.

[Note B] Calculated based on a basal level of 784 Gal80p/cell [8], the basal transcription level calculated from [6], the protein half-life from [4], and a translation rate of 4/mRNA/min.

[Note C] Calculated from estimated mRNA abundance based on [6] and mRNA half-life measurement in galactose [3].

[Note D] Assigned based on estimated average translation rate [8, 4, 9] in yeast. Inaccuracies in this rate constant are accounted for during the gene network model fitting process, by the scale parameter S_1.

[Note E] Arbitrarily assigned. Inaccuracies in this rate constant are accounted for during the fluorescence fitting process.

[Note F] Arbitrarily assigned. Inaccuracies in this rate constant are accounted for during the model fitting process, by the scale parameter S_1.

[Note G] The degradation rate is assumed to be similar to that of GAL1 mRNA. Inaccuracies in this rate constant are accounted for during the fluorescence fitting process.

[Note H] This protein is highly stable and assigned a half-life of 24 hours.

[Note I] Arbitrarily assigned (see Methods).

[Note J] Assigned based on the results from running the volume model.

References

1. Acar M, Mettetal JT, van Oudenaarden A. Stochastic switching as a survival strategy in fluctuating environments. Nat Genet. 2008;40(4):471-5. doi:10.1038/ng.110.
2. Peng W, Liu P, Xue Y, Acar M. Evolution of gene network activity by tuning the strength of negative-feedback regulation. Nat Commun. 2015;6. doi:10.1038/ncomms7226.
3. Munchel SE, Shultzaberger RK, Takizawa N, Weis K. Dynamic profiling of mRNA turnover reveals gene-specific and system-wide regulation of mRNA decay. Molecular Biology of the Cell. 2011;22(15):2787-95. doi:10.1091/mbc.E11-01-0028.
4. Belle A, Tanay A, Bitincka L, Shamir R, O'Shea EK. Quantification of protein half-lives in the budding yeast proteome. Proceedings of the National Academy of Sciences of the United States of America. 2006;103(35):13004-9. doi:10.1073/pnas.0605420103.
5. Zacharioudakis I, Gligoris T, Tzamarias D. A Yeast Catabolic Enzyme Controls Transcriptional Memory. Current Biology. 2007;17(23):2041-6. doi:10.1016/j.cub.2007.10.044.
6. Hsu C, Scherrer S, Buetti-Dinh A, Ratna P, Pizzolato J, Jaquet V et al. Stochastic signalling rewires the interaction map of a multiple feedback network during yeast evolution. Nat Commun. 2012;3:682. doi:10.1038/ncomms1687.
7. Timson DJ, Ross HC, Reece RJ. Gal3p and Gal1p interact with the transcriptional repressor Gal80p to form a complex of 1:1 stoichiometry. Biochem J. 2002;363(3):515-20.
8. Ghaemmaghami S, Huh W-K, Bower K, Howson RW, Belle A, Dephoure N et al. Global analysis of protein expression in yeast. Nature. 2003;425(6959):737-41. doi:10.1038/nature02046.
9. To T-L, Maheshri N. Noise Can Induce Bimodality in Positive Transcriptional Feedback Loops Without Bistability. Science. 2010;327(5969):1142-5. doi:10.1126/science.1178962.
